# Explainable Security in SDN-Based IoT Networks

**DOI:** 10.3390/s20247326

**Published:** 2020-12-20

**Authors:** Alper Kaan Sarica, Pelin Angin

**Affiliations:** Department of Computer Engineering, Middle East Technical University, Ankara 06800, Turkey; kaan.sarica@metu.edu.tr

**Keywords:** SDN, security, machine learning, 5G, IoT, intrusion detection

## Abstract

The significant advances in wireless networks in the past decade have made a variety of Internet of Things (IoT) use cases possible, greatly facilitating many operations in our daily lives. IoT is only expected to grow with 5G and beyond networks, which will primarily rely on software-defined networking (SDN) and network functions virtualization for achieving the promised quality of service. The prevalence of IoT and the large attack surface that it has created calls for SDN-based intelligent security solutions that achieve real-time, automated intrusion detection and mitigation. In this paper, we propose a real-time intrusion detection and mitigation solution for SDN, which aims to provide autonomous security in the high-traffic IoT networks of the 5G and beyond era, while achieving a high degree of interpretability by human experts. The proposed approach is built upon automated flow feature extraction and classification of flows while using random forest classifiers at the SDN application layer. We present an SDN-specific dataset that we generated for IoT and provide results on the accuracy of intrusion detection in addition to performance results in the presence and absence of our proposed security mechanism. The experimental results demonstrate that the proposed security approach is promising for achieving real-time, highly accurate detection and mitigation of attacks in SDN-managed IoT networks.

## 1. Introduction

The number of connected devices and Internet of Things (IoT) use cases have been continuously increasing, thanks to the developments in the fields of mobile networks, big data, and cloud computing. IoT use cases that significantly facilitate our daily lives include smart homes, autonomous cars, security systems, smart cities, and remote healthcare, among many others. When the large volumes of data generated by IoT are considered, it is obvious that the quality of service (QoS) requirements of these various use cases will not be satisfiable by legacy wireless networks. 5G and beyond networks that rely on software-defined networking (SDN) and network function virtualization (NFV) for resource management will be a key enabler for the future’s ubiquitous IoT.

IoT has already resulted in a large attack surface, due to limited processing power and battery life, as well as the lack of security standards, which make a large number of IoT devices incapable of implementing even basic security mechanisms, like encryption. New use cases, protocols, and technologies add new attack surfaces to the existing ones. It is of utmost importance to develop intrusion detection and prevention systems for IoT networks that address new and existing vulnerabilities in order to ensure the healthy operation of these systems. It is also essential to ensure the compliance of the developed security techniques with SDN-based network architectures and benefit from the network programmability that is provided by SDN to ensure fast detection and mitigation of attacks, as well as a quick reconfiguration of the networks in order to prevent QoS degradation and failures.

Machine learning (ML) techniques have become popular tools for network intrusion detection tasks in the past two decades, especially due to the increasing accuracy that is achieved by a variety of models, and the superiority that they have over rule-based systems in detecting previously unseen attacks. The developments in the field of deep learning have made them indispensable parts of any classification task, including intrusion detection. Although deep learning models have been shown to be quite successful in intrusion detection, they are usually used as blackboxes, and their decision-making processes are not readily explainable to human experts [1]. The explainability problem is especially important in the security domain [2] in order to correctly interpret the results produced by these models.

Despite the importance of realistic network traffic data for effective model building, most of the existing research in IoT intrusion detection has used datasets that were generated for legacy networks without IoT traffic. This is mostly due to the lack of publicly available datasets that include IoT traffic, except the recently released Bot-IoT dataset [3]. To the best of our knowledge, there is no publicy available dataset specifically for SDN-based IoT environments. The network traffic characteristics of IoT and SDN are quite different from those of legacy networks; therefore, using models that were trained with legacy network data might lead to inaccurate classification results. Furthermore, it is crucial for intrusion detection systems to retrieve features in real time for effective attack detection and mitigation. Existing public datasets have been created by processing pcap files and there is no guarantee that all of the features that they include can be retrieved in real time.

In an effort to address the abovementioned shortcomings of existing security approaches for SDN-based next generation mobile networks, this paper presents a real-time intrusion detection and mitigation solution for SDN, which aims to provide autonomous security in the high-traffic IoT networks of the 5G and beyond era, while achieving a high degree of interpretability by human experts. The proposed approach is built upon automated flow feature extraction and classification of flows using random forest classifiers at the SDN application layer. This allows for the detection of various classes of attacks and it takes appropriate actions by installing new flow rules. We present a SDN-specific dataset that we generated for an IoT environment and provide the results on the accuracy of intrusion detection as well as performance results in the presence and absence of our proposed security mechanism.

The rest of this paper is organized as follows: Section 2 reviews related work in intrusion detection for SDN-based networks and existing ML datasets for network intrusion detection. Section 3 provides a brief background on SDN and classification using random forest. Section 4 describes our proposed end-to-end intrusion detection and mitigation approach for SDN-based networks. Section 5 describes our public intrusion detection dataset for SDN-based IoT. Section 6 provides a detailed performance evaluation of the proposed security approach with the generated SDN dataset. Section 7 concludes the paper with future work directions.

## 2. Related Work

Intrusion detection and mitigation in networks has become an ever more important topic of research with the increasing cyber security incidents, caused by the large attack surfaces that are created by IoT. Rathore and Park [4] proposed a fog-based semi-supervised learning approach for distributed attack detection in IoT networks. The authors used the NSL-KDD dataset and showed their distributed approach performed better than centralized solutions in terms of detection time and accuracy. Evmorfos et al. [5] proposed an architecture that uses Random Neural Networks and LSTM in order to detect SYN flooding attacks in IoT networks. The authors generated their dataset by creating a virtual network and recorded the traffic into pcap files. Soe et al. [6] proposed a sequential attack detection architecture that uses three machine learning models for IoT networks. The authors used the N-BaIoT dataset and achieved 99% accuracy. Alqahtani et al. [7] proposed a genetic-based extreme gradient boosting (GXGBoost) model that uses Fisher-score in order to select features in IoT networks. The authors also used the N-BaIoT dataset and achieved 99.96% accuracy. Even though these approaches were shown to successfully detect attacks in IoT networks, their design was performed according to legacy network infrastructures that do not utilize SDN. SDN-based networks have important differences both in terms of operation and the packet flow features that can be extracted in real time, requiring compatible models to be built, as will be explained in Section 3.1.

With the increasing adoption of SDN-based network architectures in the past decade, SDN security has become one of the centers of attention for the cyber security research community. The majority of the solutions that have been proposed for SDN-based networks so far have focused on techniques for the detection and mitigation of denial-of-service (DoS) and distributed denial-of-service (DDoS) attacks. In [8], a semi-supervised model was used to detect DDoS attacks in SDN-based IoT networks. The model achieved above 96% accuracy on the UNB-ISCX dataset and the own dataset of the authors, which only included UDP flooding attacks. In [9], an entropy-based solution was proposed for the detection and mitigation of DoS and DDoS attacks in software-defined IoT networks. The approach achieved high accuracy on the Bot-IoT dataset and the authors’ dataset containing TCP SYN flooding attacks. Yin et al. [10] proposed using cosine similarity of packet_in rates received by the controller and drop packets if a predefined threshold is reached. Their approach only mitigated DDoS attacks. Ahmed and Kim [11] proposed an inter-domain information exchange approach that uses statistics that are collected from switches across different domains to mitigate DDoS attacks. Bhunia and Gurusamy [12] used Support Vector Machine (SVM) in order to detect and mitigate DoS attacks in SDN-based IoT networks. The authors created their own data; however, the dataset is not publicly available. Sharma et al. [13] proposed using deep belief networks to mitigate DDoS attacks in SDN-based cloud IoT. Bull et al. [14] used an SDN gateway to detect and block anomalous flows in IoT networks. Their approach managed to succesfully detect and mitigate TCP and ICMP flooding attacks. In spite of the fact that most of these approaches have accomplished successful detection and mitigation, they only work against DoS and DDoS attacks.

Other works have targeted coverage of additional attacks, but used datasets that are not specific to SDN for evaluation. Li et al. [15] proposed using the BAT algorithm for feature selection and then used the random forest algorithm on the KDD CUP’99 dataset, achieving 96% accuracy. In [16], the CART decision tree algorithm was proposed in order to detect anomalies in IoT networks using SDN. The authors used the CICIDS’2017 dataset and achieved a 99% detection rate. Dawoud et al. [17] proposed an SDN-based framework for IoT that uses Restricted Boltzmann Machines to detect attacks. The authors achieved a higher detection rate than existing works on the KDD CUP’99 dataset. Al Hayajneh et al. [18] proposed a solution for detecting man-in-the-middle attacks against IoT in SDN. Their solution only works for IoT devices that use HTTP for communication. Shafi et al. [19] proposed a fog-assisted SDN-based intrusion detection system for IoT that uses Alternate Decision Tree. The authors used the UNSW-NB15 dataset and achieved high detection rates. Derhab et al. [20] proposed an intrusion detection system, which uses Random Subspace Learning, K-Nearest Neighbor and blockchain against attacks that target industrial control processes. The authors used the Industrial Control System Cyber attack dataset and demonstrated their solution achieves high accuracy. Work in explainable intrusion detection systems has been rather limited so far. One example is the work of Wang et al., who proposed an explainable machine learning framework for intrusion detection systems that are based on Shapley Additive Explanations [21]. The framework was evaluated on the NSL-KDD dataset and it achieved promising results.

Network intrusion detection using ML techniques has been a popular approach of network security, especially for the past two decades, for which researchers have created a number of extensive network trace datasets. These datasets, even if they are old, are still in use today by security researchers as benchmarks. Among existing publicly available network intrusion detection datasets are the following:

- **KDD CUP’99** [22] was generated in 1999 by extracting features from the DARPA98 [23] dataset, which simulates a U.S. Air Force LAN. KDD CUP’99 has 41 features and four attack categories: DoS, R2L, U2R and probing. Even though it is an old dataset, many researchers still use this dataset. However, it is not without some drawbacks. Firstly, the distribution of the records in the training and test sets are widely different, because the test set includes some attack types that are not in the training set [24]. Secondly, around 75% of the data in the training and test sets are duplicates [24], which could lead to biased classification models. Most importantly, the dataset was not generated in an IoT environment and it does not include SDN-specific features.
- **NSL-KDD** [24] was created to improve the KDD CUP’99 dataset. Duplicate records were eliminated and the number of records was reduced. Also classes were balanced. Still, this dataset does not represent the behavior of current networks.
- **UNB-ISCX** [25] was created by the Canadian Institute of Cybersecurity in 2012. Real network traces were analyzed to create realistic profiles. The dataset consists of seven days of network traffic containing three types of attacks: DDoS, brute force SSH, and infiltrating the network from inside.
- **CAIDA** [26] contains anonymized network traces. Records were created by removing the payloads of the packets and anonymizing the headers. This dataset only contains DoS attacks and features are the header fields. Additional features using the header fields were not generated.
- **UNSW-NB15** [27] was created in 2015. The IXIA tool was used to generate the network traffic. UNSW-NB15 has 49 features and two of them are labels for binary and multi-class classification. The dataset consists of normal traffic and nine types of attack traffic, namely DoS, DDoS, fuzzing, backdoor, analysis, exploit, generic, worm, and shellcode. The main problem of the dataset is the lack of sufficiently many samples for some attack types.
- **CICIDS2017** [28] is another dataset that was created by the Canadian Institute of Cybersecurity. Realistic benign traffic was created using their B-Profile system. The dataset includes normal traffic and six types of attack traffic, namely DoS, botnet, port scanning, brute force, infiltration, and web attack.
- **Bot-IoT** [3] was introduced in 2018. The most important feature of the dataset is that it includes IoT traffic, unlike most of the existing intrusion detection datasets. The dataset has 46 features and two of them are labels for binary and multi-class classification. The dataset consist of normal traffic and six different attack types, namely DoS, DDoS, service scanning, OS fingerprinting, data theft, and keylogging. The main problem of the dataset is the lack of sufficiently many samples for some attack types. The number of records for normal traffic is also low.

Most of the existing work on intrusion detection systems for IoT and SDN environments used the datasets that are mentioned above. However, these datasets were not created in networks managed by SDN. Furthermore, these datasets do not contain IoT traffic, except for the BoT-IoT dataset. Most of the existing datasets were created by recording and processing pcap files with different tools. Therefore, an SDN controller may not be able to obtain all of the features in real time. To the best of our knowledge, there is no other publicly available SDN dataset that includes IoT traffic.

## 3. Preliminaries

This section provides an overview of SDN and the random forest classifier, which are key components of the proposed solution.

### 3.1. Software-Defined Networks (SDN)

Software-defined networking (SDN) emerged as a novel networking paradigm in the past decade, supporting the need for programmatically managing networks, the operational costs of which were increasing sharply with the widespread use and new technologies that are needed to accommodate various IoT use cases. SDN differs from traditional networks by separating the data and control planes, where routers/switches are now responsible for forwarding functionality, where routing decisions are taken by the controller (control plane).

**Figure 1:**
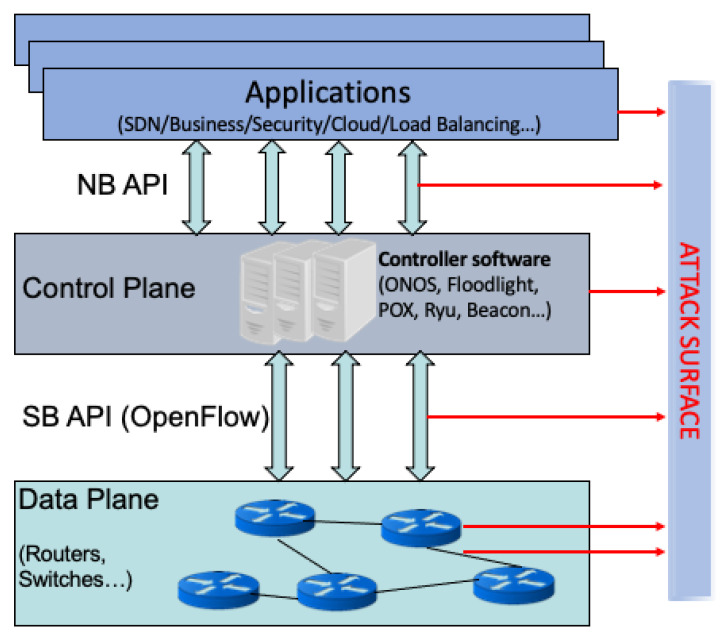
Software-defined networking (SDN) Architecture and Attack Surface.

The SDN architecture mainly consists of three layers: applications, control, and infrastructure (data plane), as seen in Figure 1. All of the applications, such as load balancing and intrusion detection systems, run on the application layer and communication with the controller takes place through the north-bound API. Communication between the controller and switches takes place through the south-bound API, mainly using the OpenFlow [29] protocol. The logically centralized controller is responsible for managing the network. The controller maintains a global view of the network and installs forwarding rules, called “flow rules”, into the corresponding switches based on the routing decisions it makes. Switches store flow rules in their flow tables and forward network packets based on matches with existing flow rules.

**Figure 2:**
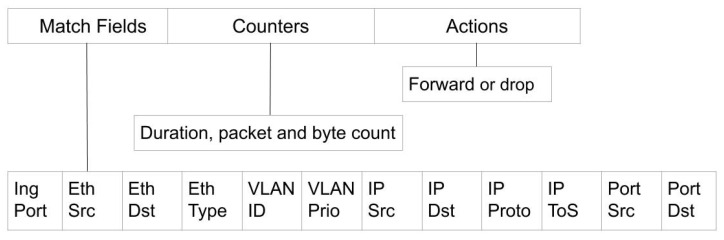
Flow Rule Structure.

Figure 2 shows the structure of a flow rule. It is mainly composed of three parts: match fields, counters, and actions. Unlike traditional networks that perform forwarding based on the destination addresses, match fields are determined by the configuration of the forwarding application and might be ingress port, VLAN ID, source and/or destination MAC addresses, IP addresses, and/or port numbers. Counters keep track of the duration of the flow and byte and packet counts that matched the flow. The action can be forwarding the packet to the specified port or dropping it, among others.

The header fields of incoming packets are compared with the match fields of flow rules in the switch. All of the required header fields of the packet should match with the match fields of a rule in the flow table to be forwarded immediately. Otherwise, the switch will buffer the packet and send what is called a *packet_in* message to the controller that contains the header fields of the packet. The controller then examines the packet_in message and generates a routing decision for the packet, which is sent back to the switch in a *packet_out* message. The necessary action is taken for the packet and the corresponding flow rules are installed to the switches by sending *flow_mod* messages. Even though the controller decides to install flow rules into the corresponding switches, a packet_out message is always sent before the flow_mod message. Therefore, unlike traditional networks, the statistics of the first packet that triggered flow rule installation cannot be seen in the installed flow rule. For TCP connections, statistics of the SYN and SYN ACK packets are lost, because they are the first packets sent from source to destination and destination to source, respectively. During DoS and DDoS attacks with spoofed addresses, all of the incoming packets may have different source addresses. Therefore, all of the incoming packets from the attacker may trigger a new flow rule installation.

#### SDN in 5G Networks

While early adoptions of SDN mostly took place in wired enterprise networks, its flexibility, programmability, speed, and cost advantages have recently made it a promising tool for other networks, including wireless sensor networks (WSNs) [30] and next generation wireless networking infrastructures. SDN will be one of the greatest enablers of 5G and beyond networks by providing the network virtualization capabilities that are needed to remotely and dynamically manage the networks. The fast failover and autonomous management capabilities to be achieved with SDN applications will provide the high bandwidth and low delay requirements of 5G networks, making them support a variety of IoT use cases. SDN, together with network functions virtualization (NFV), will especially form the basis of network slicing in 5G core and radio access networks, which will be a significant enabler for operators to efficiently utilize their infrastructure in order to provide the required quality of service and security guarantees to their customers [31].

**Figure 3:**
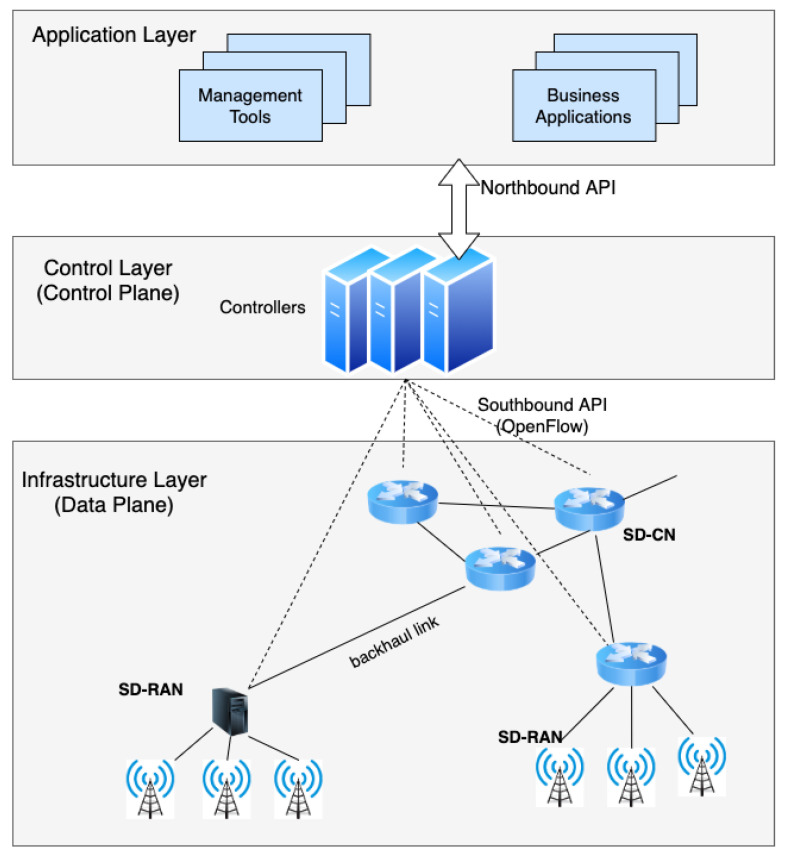
Software-Defined 5G Network Architecture.

A number of SDN-based architectures for 5G networks have been proposed [32]. One of the early proposals is SoftAir by Akyildiz et al. [33], where the data plane is a programmable network forwarding infrastructure that consists of software-defined core network (SD-CN) and software-defined radio access network (SD-RAN). While SD-RAN contains software-defined base stations, including small cells (microcells, femtocells, and picocells) in addition to traditional macro cells, SD-CN contains software-defined switches that form the 5G core network, as seen in Figure 3. User equipment and other devices are connected to the software-defined base stations or wireless access points, which are connected to the software-defined core network through the backhaul links. As proposed in SoftAir, SD-CN and SD-RAN can both use OpenFlow as the southbound API, which will provide a uniform interface with the controller routing traffic from the base stations through the optimal paths in the core network. This architecture enables the application of many of the same principles in terms of network control from wired SDN to SDN-based 5G networks.

One of the biggest promises of and reasons for the introduction of SDN is the provisioning of improved security in the network through global visibility, and the fast automated reconfiguration of flow rules. This will enable real-time detection and mitigation of malicious traffic in the network. As seen in Figure 1, an SDN can be attacked at various surfaces (the attack surface is demonstrated by red arrows pointing out from the devices, applications, or interfaces that could be attacked in SDN). These attacks could not only target the data plane devices, but also the controller and applications to cause disruptions in network operation. In this work, we focus on attacks that affect the data and control planes and propose an intrusion detection and mitigation solution that provides automated responses to attacks detected while using highly interpretable ML algorithms that are described in the next section.

### 3.2. Random Forest Classifier

Random forest (RF) is a machine learning model that constructs an ensemble of decision trees, named a forest, such that each decision tree is constructed using an independently and identically distributed random vector [34]. For classifying a particular data instance, a random forest uses the outputs of all trees in the forest to pick the majority decision. The utilization of the outputs of multiple trees makes the classifier more robust than decision trees, which suffer from the overfitting problem in many cases.

At a high level, the RF algorithm works as follows:

1. The complete training set *S* consisting of *n* data instances with class labels {$c_{i}$, i = 1, …, *n*} from a set of classes *C* is split into *k* random subsets using bootstrap sampling:
  (1) $$S=S_{1},S_{2},\ldots ,S_{k}$$
2. A random feature vector $\theta_{i}$ is created and used to build a decision tree from each $S_{i}$. All  {$\theta_{i}$, *i* = 1, 2, 3, …, $\theta_{k}$} are independent and identically distributed.
3. Each tree r($S_{i}$, $\theta_{i}$) is grown without pruning to form the forest R.
4. The classification of a test data instance *x* is calculated, as follows:
  (2) $$H\left(x\right)=max_{C_{j}}\sum_{i=1}^{k}\left(I\left(h_{i}\left(x\right)=C_{j}\right)\right)$$
  where *I* is the indicator function and $h_{i}\left(x\right)$ is the result of classification by $r(S_{i},\theta_{i})$.

**Figure 4:**
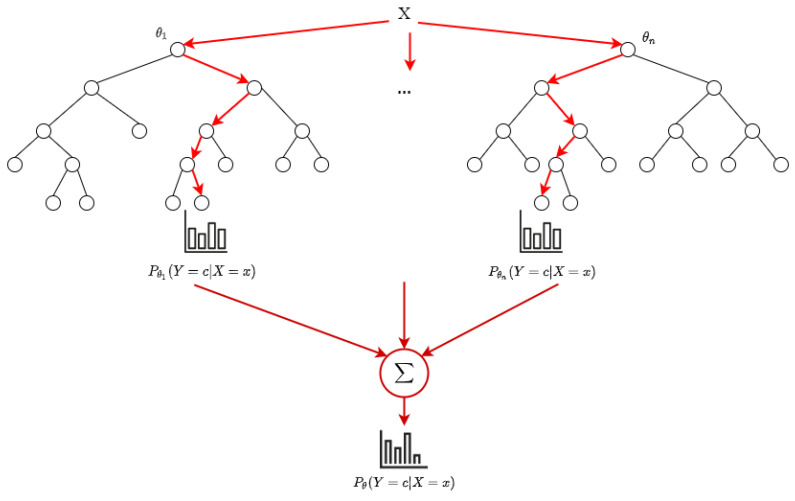
Classification by Random Forest.

Figure 4 shows a simplified view of classification by random forests. Here, the child branches of the root show the different trees in the random forest. When a data item *X* needs to be classified, its probability of belonging to class *c* is calculated as the sum of the class probabilities for each decision tree $\theta_{i}$ ($\theta_{1}$…$\theta_{n}$ in the figure), averaged over all trees. The item will then be assigned to the class with the highest probability. The nodes in each decision tree here use binary splits that are based on a specific feature value (e.g., is number of bytes ≤ 118?), and the branches of the tree are followed up until the leaves by checking the values of those features in data item *X*, as depicted by the red arrows pointing towards child nodes from the internal nodes of the trees.

Information gain is a commonly used metric for deciding the splitting criteria for the various nodes in the decision trees. The information gain from the split of a node *S* based on a random variable *a* is calculated as follows:

(3) IG(S,a)=E(S)−E(S|a)

Here, E(S) is the entropy of the parent node before the split and *E*(*S*|*a*) is the weighted average of the entropies of the child nodes after the split. E(S) is calculated as:

(4) $$E\left(S\right)=-\sum_{i=1}^{C}p\left(c_{i}\right)logp\left(c_{i}\right)$$

where $p(c_{i})$ is the probability of a data instance in node S having class label $c_{i}$.

**Figure 5:**
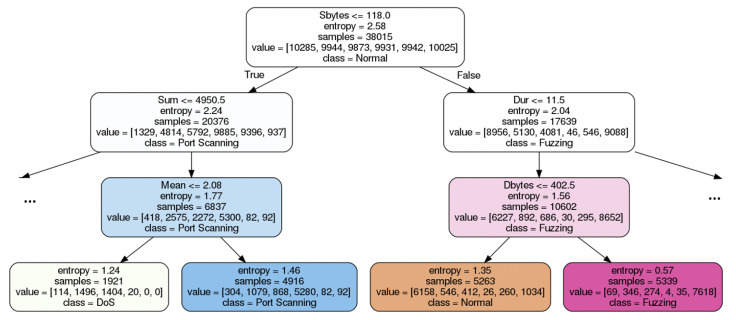
Sample Decision Tree from Random Forest for a Network Intrusion Detection Task.

Figure 5 shows a partial view of a decision tree from the random forest constructed for a sample network intrusion detection task on the IoT network dataset that we have generated. As seen in the figure, the entropy of nodes decreases while approaching the leaves, as nodes that are higher up in the tree are split based on a specific threshold of feature values discovered by the algorithm. A random forest contains a multitude of such decision trees, each constructed from a different, randomly sampled subset of the whole training data.

RF is among the ML algorithms with the highest degree of explainability/interpretability, due to its reliance on decision trees, which construct models based on splits of training data along feature values, which are easily readable by human domain experts. The effectiveness of RF for a variety of classification tasks has been shown in many studies [35]. Despite the success of especially deep learning algorithms in various classification tasks in recent years, RF continues to outperform many state-of-the-art ML algorithms, especially in tasks that involve structured data.

## 4. Proposed Security Approach

**Figure 6:**
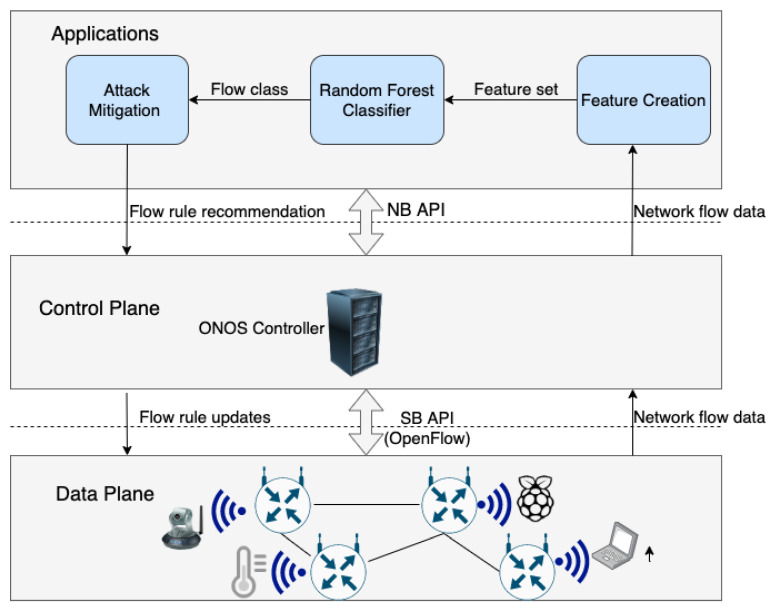
SDN-based Security Solution Architecture.

The proposed intrusion detection and mitigation approach, the overall operation of which is depicted in Figure 6, provides security in SDN-based networks by automated, intelligent analysis of network flows, followed by mitigation actions being taken in accordance with the decision of the intrusion detection component. The end-to-end intrusion detection and mitigation process relies on three main applications in the application layer, namely Feature Creator, RF classifier, and Attack Mitigator.

The Feature Creator collects network flows from the switches at regular intervals and calculates the values of features that are required by the RF classifier for each flow. The RF classifier applies its pre-built intrusion detection model on the flow instance and passes the result to the Attack Mitigator. The Attack Mitigator then determines the action to take based on the classification result and installs flow rules into the corresponding switches to mitigate the attack if necessary. Algorithm 1 summarizes the end-to-end operation of the proposed security solution.

**Algorithm 1** End-to-end Operation
$L_{S}$← Get connected SDN switches*M*← Load RF classifier model$L_{B}$← Blacklist**while** True **do**  **for all** *S* in $L_{S}$ **do**    $L_{E}$← Pull flow entries from *S*    *F*← FEATURE_CREATION($L_{E}$)    ATTACK_DETECTION(*F*)  **end for**  Wait for some time
**end while**
**procedure**Attack_Detection(*F*)  *C*← Classify *F* using *M*  **if** *C* is attack **then**    *I*← Get source identifiers from *F*    MITIGATION(C,I)  **end if**
**end procedure**
**procedure**Mitigation(C,I)  **if** *I* is not in $L_{B}$ **then**    *E*← Create flow entry to block or redirect *I*    Install *E* into *S*    $L_{B}$.add(*I*)  **end if**
**end procedure**



The controller periodically collects network flow entries from the switches, which are retrieved by the Feature Creator at regular intervals. Upon retrieval, features are created for every flow, as summarized in Algorithm 2. Common features for every flow are generated, looping over every flow entry in the switch using Algorithm 3. e.g., the average duration of flows and total number of packets in a transaction are created with an initial pass over flow entries. Subsequently, flow-specific features, e.g., the duration of flow and source-to-destination packet count, are retrieved by passing over all of the flow entries. While looping over flow entries, the created feature vector for a flow is immediately sent to the RF classifier, without waiting to finish feature creation for other flow entries. The Feature Creator also retrieves flow match fields, like source IP and MAC addresses, and the physical port of the switch where the packet is coming from. The Attack Mitigator uses these features.

**Algorithm 2** Feature Creation
**procedure**Feature_Creation(Flow entries)  $L_{E}$← Flow entries  *F*← Feature vector  *C*← CALCULATE_COMMON($L_{E}$)  **for all** *E* in $L_{E}$ **do**    F.Sbytes←*E*.getByteCount()    F.Spkts←*E*.getPacketCount()    F.Dur←*E*.getDuration()    F.Mean←C.mean    F.Stddev←C.stddev    F.Sum←C.sum    F.TnP_PSrcIP←C.TnP_PSrcIP    F.TnP_PDstIP←C.TnP_PDstIP    F.TnP_Per_Dport←C.TnP_Per_Dport    srcIP←*E*.getSourceIp()    dstIP←*E*.getDestinationIp()    *P*←*E*.getSwitchPort()    proto_number←*E*.getInternetProtocolNumber()    **if** proto_number is TCP or UDP **then**      srcPort←*E*.getSourcePort()      dstPort←*E*.getDestinationPort()      key← dstIP + dstPort + srcIP + srcPort      F.Dbytes←C.hashMap.get(key)    **else if** proto_number is ICMP **then**      key← dstIP + srcIP      F.Dbytes←C.hashMap.get(key)    **end if**  **end for**   return F  
**end procedure**



The common features include Mean, Stddev, Sum, TnP_PSrcIP, TnP_PDstIP, and TnP_Per_Dport. Their detailed descriptions can be found in Table 1. Hash sets are used to store unique source IPs, destination IPs, and destination port numbers. A list is used to store the duration of flow entries. While looping over the flow entries, packet counts of the flow entries are added to the total packet count. The duration of the flow entries are added to the duration list. Source IPs, destination IPs, and destination port numbers are added to the corresponding hash sets. Byte counts of the flows are added to a hash map. Keys of this map are made of source IP, source port, destination IP, and destination port for TCP and UDP packets. For ICMP packets, the keys are made of source IP and destination IP, since they do not have port numbers. This map is later used for retrieving reverse flow statistics. After looping over all of the flow entries, common features are calculated using the total packet count, hash sets, and duration list. Flow-specific features, i.e., Dur, Spkts, Sbytes, and Dbytes, are calculated within the second pass over the flow entries. Duration, packet count, and byte count of the flow entries are extracted. The hash map that was created in the common feature creation is used to retrieve destination-to-source byte count. After creating the feature vector for a flow entry, it is sent for classification without waiting for the creation of other feature vectors.

**Algorithm 3** Calculation of common statistics and features
**procedure**Calculate_Common($L_{E}$)  *C*← Common statistics  srcIpSet← Create source IP HashSet  dstIpSet← Create destination IP HashSet  portSet← Create destination port HashSet  $L_{D}$← Create duration List  totalPacketCnt← 0  **for all** *E* in $L_{E}$ **do**    pkts←*E*.getPacketCount()    totalPacketCnt←totalPacketCnt + pkts    bytes←*E*.getByteCount()    $L_{D}$.add(*E*.getDuration())    srcIP←*E*.getSourceIp()    srcIpSet.add(srcIP)    dstIP←*E*.getDestinationIp()    dstIpSet.add(dstIP)    proto_number←*E*.getInternetProtocolNumber()    **if** proto_number is TCP or UDP **then**      srcPort←*E*.getSourcePort()      dstPort←*E*.getDestinationPort()      key← srcIP + srcPort + dstIP + dstPort      C.hashMap.put(key,bytes)    **else if** proto_number is ICMP **then**      key← srcIP + dstIP      C.hashMap.put(key,bytes)    **end if**  **end for**  C.tnP_PSrcIp←totalPacketCnt/srcIpSet.size()  C.tnP_PDstIp←totalPacketCnt/dstIpSet.size()  C.tnP_Per_DPort←totalPacketCnt/portSet.size()  C.mean← mean of $L_{D}$  C.stddev← standard deviation of $L_{D}$  C.sum← summation of $L_{D}$  **return** *C* 
**end procedure**



The RF classifier, which works as explained in Section 3, gets feature vectors from the Feature Creator one-by-one and classifies them using its pre-built intrusion detection model. If the outcome of the classification is any attack type, the Attack Mitigator is sent the detected attack type and source identifiers, i.e., source IP, source MAC address, and the physical switch port that the packet is coming from. The used machine learning model should be updated dynamically by the inclusion of new training data for existing attack types or adding new attack types as they are discovered. The RF model built is a multi-class classification model that is formed using training data that consists of various attack types in addition to normal traffic. We advocate using multi-class attack classification rather than binary classification/anomaly detection, as the former provides more informed decision-making capability in terms of the action to take/the specific flow rule to install.

As discussed previously, the RF classifier creates results that are highly explainable to human experts, as opposed to blackbox ML models, whose results are not easily interpretable. For instance, when a specific flow is classified as a DoS attack, it is possible to trace the trees in the forest that voted as a DoS and which feature values caused them to make that decision. This provides the ability for a human network expert to judge the quality of the model, provide recommendations, update the model, or take additional actions if necessary.

The Attack Mitigator is informed by the RF classifier upon attack detection. This component creates a flow rule update recommendation, depending on the attack type. The created rule update is sent to the controller, which installs the flow entries into the corresponding switches. The installed flow entries have higher priority than normal flow entries in the switch. The corresponding action can be dropping the matching packets or redirecting matching flows to a honeypot. Packet blocking and redirection can be based on the source MAC address, source IP, or the physical switch port.

## 5. SDN Datasets

In this section, we provide details of our SDN-based IoT network datasets that were generated based on the packet sending rates and packet sizes from an IoT dataset generated in a real testbed. All of our features are SDN-specific and they can be retrieved using an SDN application in real time. The accuracy of the RF classifier was evaluated with the two publicly available SDN datasets we generated and compared with the accuracies of state-of-the-art ML algorithms. Feature selection was used to identify important features for detecting attacks in SDN-based IoT networks. The performance of the model was also evaluated under network changes.

We have created 2 SDN datasets [36] and made them available online [37]. Their only difference is the number of IoT devices. In IoT networks, the number of IoT devices may change over time. Our second dataset has more IoT devices and the number of active IoT devices also changed during the traffic recording. The second dataset enables us to evaluate the performance of the models trained with the first dataset. That way we can have an idea about how our model will be affected when the number of IoT devices changes and how often we should update our model. Our datasets contain normal traffic and five different attack types, namely DoS, DDoS, port scanning, OS fingerprinting, and fuzzing.

### 5.1. Testbed Overview

We used a similar network topology to the Bot-IoT dataset [3]. Mininet [38] was used to virtualize our network and an ONOS controller [39] managed the network. An Open vSwitch [40] was used to connect the controller and simulated devices.

### 5.2. Benign Traffic

Similar packet sizes and sending rates to the BoT-IoT dataset [3] were used for benign traffic. Our IoT devices simulated IoT services that send small amounts of data to a server periodically, e.g., a smart fridge or weather station. IoT devices sent one or two packets to the server at a time using TCP. We used five simulated IoT devices in our first dataset. In our second dataset, we initially had 10 IoT devices and two of them were turned off after some time during every recording. Two benign hosts in our network sent large amounts of data to the server. One of them used UDP and the other one used TCP. We generated and recorded the benign traffic both with and without the presence of malicious traffic.

### 5.3. Malicious Traffic

Up to four attacker hosts performed different types of attacks targeting the server or IoT devices, depending on the attack type. We performed five types of attacks, namely DoS, DDoS, port scanning, OS fingerprinting, and fuzzing.

- DoS: the Hping3 tool [41] was used for DoS attacks. One malicious host launched the attacks with and without spoofed IP addresses targeting the server or one of the IoT devices. Using spoofed IP addresses causes every attack packet to trigger a new flow rule installation and wastes resources of both the controller and switches. We performed both SYN flood and UDP flood attacks. All of the combinations of four packet sending rates (4000, 6000, 8000, and 10,000 packets per second) and payloads (0, 100, 500, and 1000 bytes) were used.
- DDoS: all of the four malicious hosts participated in this attack. The same scenarios as DoS were performed.
- Port scanning: the Nmap tool [42] was used for port scanning attacks. One malicious host launched the attack targeting the server or one of the IoT devices. Nmap has two options for port scanning: by default, the first 1024 ports are scanned and users can also specify the range of ports to scan. We scanned the first 1024 ports and all of the port numbers (0 to 65,535).
- OS fingerprinting: Nmap was used for the OS fingerprinting attack. During this attack, the attacker first performs a simple port scanning to detect open ports. Subsequently, the attacker uses these ports to proceed with the attack. Therefore, we used one malicious host to launch the attack only targeting the server.
- Fuzzing: Boofuzz [43] was used for fuzzing attacks. The aim of this attack is to detect vulnerabilities of the target by sending random data until the target crashes. We performed both ftp fuzzing and http fuzzing attacks using one of the malicious hosts and targeted the server. Our fuzzers know the expected input format for http and ftp connections and generated random values for input fields. For example, for http fuzzing, http methods like get, head, post, put, delete, connect, options, and trace were fuzzed with random request URI and http version fields.

### 5.4. Flow Collection and Feature Generation

Our goal was to create a dataset that can be used in the real-time detection and mitigation of malicious traffic. Therefore, unlike most of the existing datasets that are generated by recording and processing pcap files, we used an SDN application to retrieve flow entries and create our features. We configured ONOS to pull flow entries from the switches every second. Our SDN application periodically retrieved flow entries from the ONOS controller and generated our features for each flow in the switch. The SDN application waited for one second after every feature generation period and then continued to create features by retrieving new flow rules from ONOS.

Our datasets contain 33 features and Table 2 shows our features and their descriptions. Attack and category features are our labels. The attack label can be used for binary classification and the category label can be used for multi-class classification.

Every match field of the incoming packet must match with a flow rule; otherwise, a new flow rule is installed, as mentioned in the SDN section. Performing DoS and DDoS attacks using spoofed IP addresses triggered the installation of lots of duplicate flows into the switch. Therefore, we limited the number of recorded packets to 100 at each iteration of feature generation for these attack scenarios. Our first SDN dataset has 27.9 million records and the second one has 30.2 million records. Table 3 and Table 4 show the distributions of records in our datasets.

**Figure 7:**
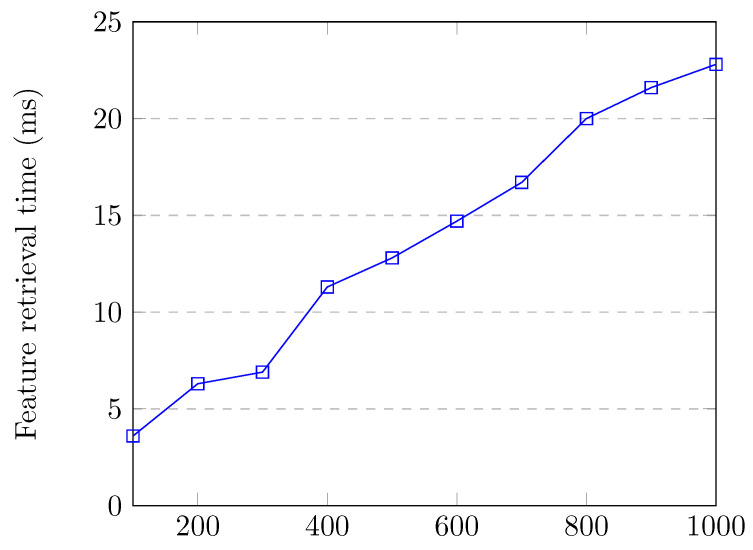
Feature retrieval time up to 1000 flow entries.

Feature retrieval time is very important, as there is no point in detecting attacks after they are over or have caused severe damage. Features should be retrieved quickly for efficient attack detection and prevention. Additionally, the feature retrieval process should not consume a lot of controller resources, otherwise network performance would be adversely affected. Figure 7 shows the flow entry collection and feature creation time up to 1000 flow entries in the switch, which corresponds to the normal traffic. When the switch had 1000 flow entries, flow collection and feature creation time for all of the flows was around 22.8 milliseconds, which is quite low.

**Figure 8:**
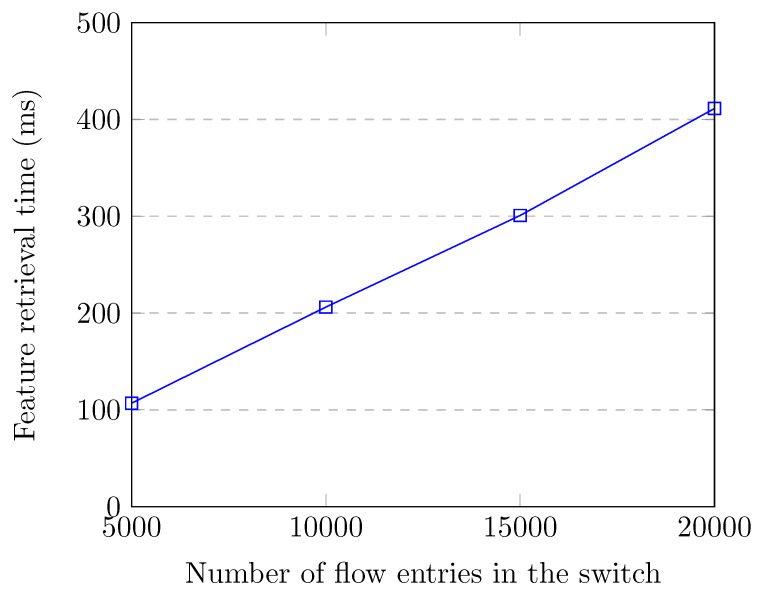
Feature retrieval time up to 20,000 flow entries.

Figure 8 shows the flow entry collection and feature creation time up to 20,000 flow entries in the switch, which corresponds to the attack traffic. Even though there were 20,000 flow entries in the switch, our SDN application collected flow entries and created features for all of the flows in 411.3 milliseconds, which does not cause much overhead for our controller. We observe that the feature retrieval time increases linearly with the number of flow entries in the switch.

### 5.5. Pre-Processing

Our datasets contain millions of records and record counts that belong to every category is not the same, as shown in Table 3 and Table 4. Processing millions of data records is not feasible and it may lead to overfitting. Additionally, imbalanced datasets might cause biased models. Around 74% of the records belong to the port scanning attack. Therefore, we wanted to take an equal number of records from every category for model training. We also wanted to take an equal number of records from each recording of a category. The reason is that, depending on the configuration and target, the record counts differed a lot. For example, one of the DoS attacks without spoofing had the lowest record count of 3251, while DoS attacks with spoofing had up to 137,000 records. We recorded DoS traffic 12 times, so the maximum number of records that we could get was 39,012. Therefore, we took 35,000 records from every attack category taken equally from every scenario of that attack type, which resulted in a total of 175,000 attack records.

For multi-class classification, we also took 35,000 normal records. Normal records were taken equally from the DoS, DDoS, port scanning, OS fingerprinting, fuzzing, and normal traffic without attack files, 5834 each. The constructed dataset had 35,000 records from every category, with a total of 210,000 records. We split this dataset into training and test sets. The training set has 25,000 records from every category, with a total of 150,000 records. The test set had 10,000 records from every category, with a total of 60,000 records.

The same procedure was followed for both of the datasets, and training and test datasets were created for both.

### 5.6. Multi-Class Classification

**Figure 9:**
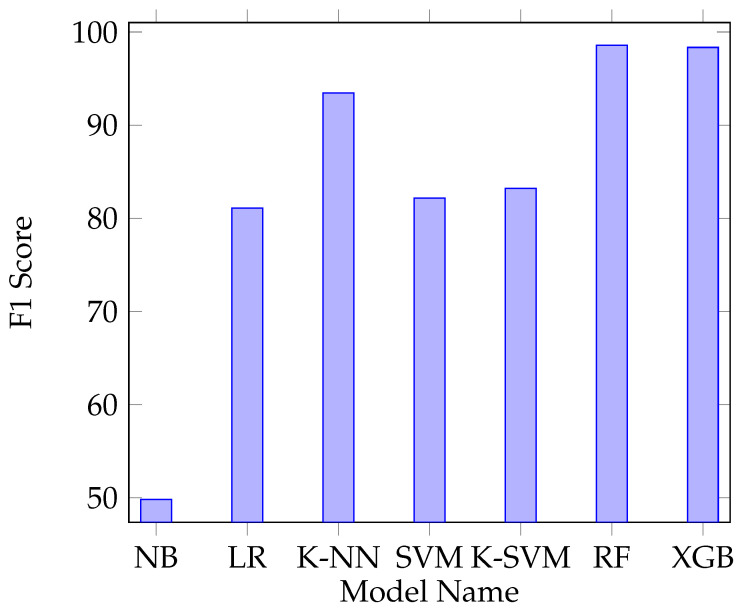
Model comparison.

The constructed training and test sets were used in order to evaluate the performances of different machine learning algorithms. We have used all of the features, except host identifiers: srcMac, dstMac, srcIP, dstIP, srcPort, dstPort, last_seen, and proto_number. Different machine learning algorithms were trained and tested using the first SDN dataset’s training and test sets. Figure 9 shows the results of multi-class classification of different algorithms: naive bayes (NB), logistic regression (LR), k-nearest neightbour (K-NN), support vector machines (SVM), kernel support vector machines (K-SVM), random forest (RF), and XGBoost (XGB). RF and XGB performed better than the other algorithms.

Our goal of creating two datasets was to perform tests on the second dataset using the models that were trained with the first dataset and see how the system would be affected from network changes. We applied feature selection based on the feature importance attribute of random forest and XGBoost algorithms. The feature importance attribute returns impurity-based feature importance of each feature in the training set. We used the features that had higher feature importance than the average of the feature importance values. We also added one feature whose importance was close to the average and ended up with 10 features. Table 5 shows the selected features and their descriptions.

The overall F1 score of the RF model, trained with the selected features, on the first dataset, was 97.86%. Performance was still close to the model trained with all of the training data and 24 features, even though we reduced both training data and the number of features by more than half. Using less features also allows for our SDN application to retrieve features much more quickly. Table 6 shows the performance metrics for all classes in the first dataset.

Normal traffic had the lowest F1 score, which is not desirable, as we do not want to classify normal packets as malicious packets and block legitimate traffic. Five over six of the normal records in our test set belonged to the normal traffic during attack scenarios. Distinguishing normal traffic from attack traffic during an attack is not an easy task. Therefore, we must be sure before taking action upon detecting an attack. In the absence of any attack traffic, our model’s accuracy of detecting normal traffic was 99.67%, which is quite high.

The overall F1 score on the second dataset was 84.48% using the initially selected 10 features. Two features were replaced and the overall performance increased to 91% using the features that are listed in Table 7 and the hyperparameters listed in Table 8. Our model’s performance for the normal traffic on the second dataset was similar to the performance on the first dataset. However, the overall performance was lower than the first dataset, because our model classified some of the DoS attacks as DDoS attacks on the second dataset as expected, due to the increased number of IoT devices in the second dataset. Because the mitigation action taken is the same, the network performance is not affected.

F1 scores for every class in the first dataset are shown in Table 9. Results are similar to the initially selected features. On the other hand, using the features in Table 7 performed well both on the first and second datasets.

## 6. Experimental Evaluation

In this section, we provide an experimental evaluation of the proposed security approach using an SDN-managed IoT network simulation environment. We performed experiments to evaluate the end-to-end intrusion detection and mitigation model in terms of its effect on the network parameters during DoS attacks of different types. The experiments were conducted on a machine with Intel Core i7-8750H @ 2.20GHz processor and 16 GB RAM.

### 6.1. Experiment Setup

**Figure 10:**
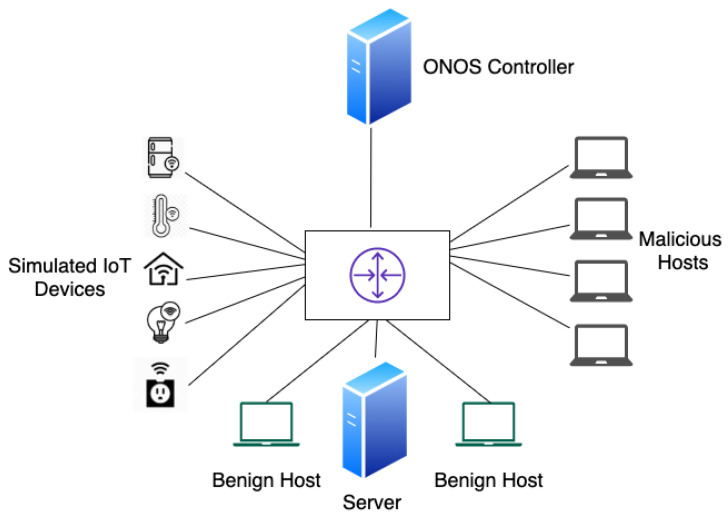
Testbed environment.

For the deployment of the proposed intrusion detection and mitigation system, the testbed setup in Figure 10 was used. Mininet was used to create a virtual network. The maximum bandwidth of each link in the network was limited to 100 Mb per second. An ONOS controller managed the network. Simulated IoT devices, benign hosts, and the server transmitted data, as explained in the SDN Datasets section.

Some attack types also affect the performance of the network as well as the target. DoS and DDoS attacks decrease the available bandwidth and consume resources of the controller and switches. Other attack types in our dataset do not have a significant effect on the network. Their purpose is to find vulnerabilities of the target and crash it if possible. Therefore, we focused on DoS and DDoS attacks in our network performance experiments. One malicious host was used to perform DoS attacks and effects of the attacks on the network were measured.

The ONOS controller was configured to pull flow entries from the switch every second. Our SDN application retrieved flow entries from the controller and generated the 10 best features that were required by our random forest classifier for each flow entry. The SDN application waited for one second after creating features for all of the flow entries in the switch and then continued to create features by retrieving new flow entries from the controller.

**Figure 11:**
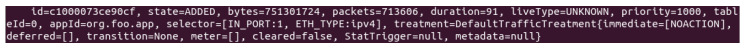
Installed flow rule for dropping packets.

**Figure 12:**
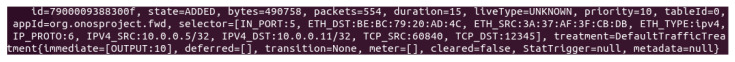
Installed flow rule for normal packets.

Our model classified every flow entry. When our application detected the third attack flow coming from a switch port, the mitigation process started. If an attack was detected, then the attacker was blocked based on the port through which it was connected to the switch through installation of a new flow rule. The installed flow rule had a priority of 1000, which is higher than the default flow rule priority (10). Figure 11 shows a flow rule installed by our application to drop the packets coming from port 1, and Figure 12 shows a flow rule for a packet classified as normal. Here, “Selector” shows the packet match fields and their values. The “Immediate” field of the “treatment” shows the action upon matched packets. If “OUTPUT” is specified, then packets are forwarded to the specified switch port. "NOACTION" means dropping the packet.

### 6.2. Network Performance Results

In the following subsections, performance measurements of our intrusion detection and mitigation system are reported.

#### 6.2.1. Time Measurements

We measured the feature retrieval time and also feature retrieval and classification time using our SDN application. The counter was started before our application pulled flow entries from the switch and stopped when feature calculation and classification was over for all of the flow entries in the switch.

**Figure 13:**
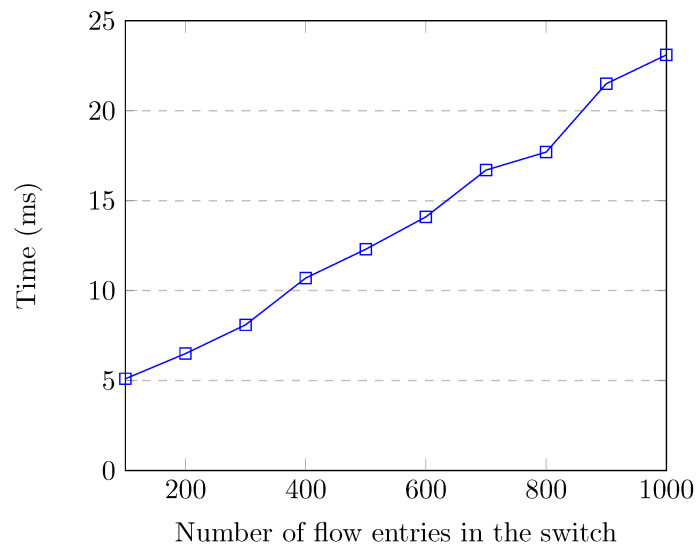
Feature retrieval time of 10 best features up to 1000 flow entries.

**Figure 14:**
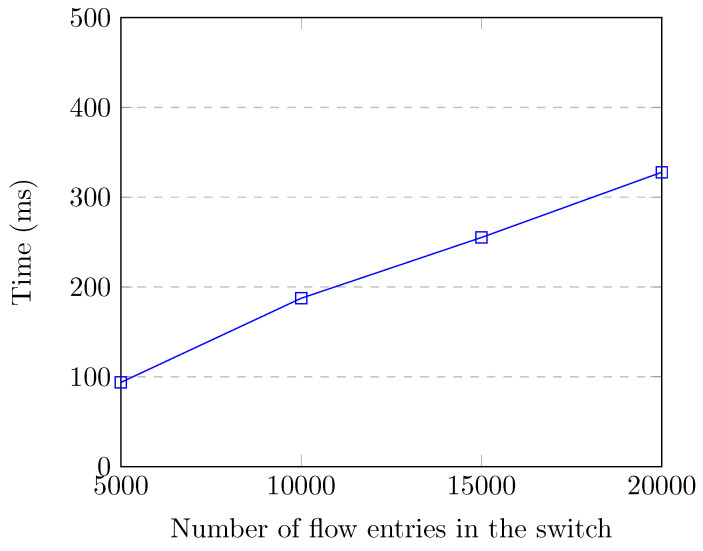
Feature retrieval time of 10 best features up to 20,000 flow entries.

Figure 13 and Figure 14 show the feature retrieval times of our 10 best features used by the RF model. Figure 13 corresponds to the network without presence of attacks. Figure 14 corresponds to the network under a DoS attack. The feature retrieval time of all features for 20,000 flow entries was 411 milliseconds, whereas it was 327 milliseconds for retrieving the 10 best features. When our application calculates the common features, it also creates a hash map that uses source IP, source port, destination IP, and destination port as the key and byte count, packet count, and packet rate as values. This map is later used for obtaining reverse flow statistics, i.e., destination-to-source packet count, byte count, and packet rate. Converting source and destination IP to a string for the key of the map takes a long time. Our model uses destination-to-source byte count (Dbytes) as a feature, as shown in Table 7. This is the reason why improvement on the feature retrieval time was not much.

**Figure 15:**
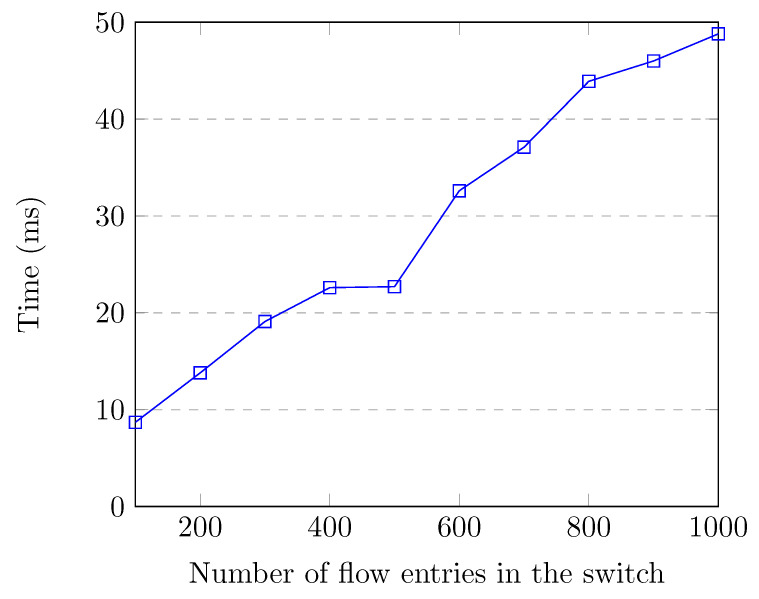
Feature retrieval time of 10 best features and classification time up to 1000 flow entries.

**Figure 16:**
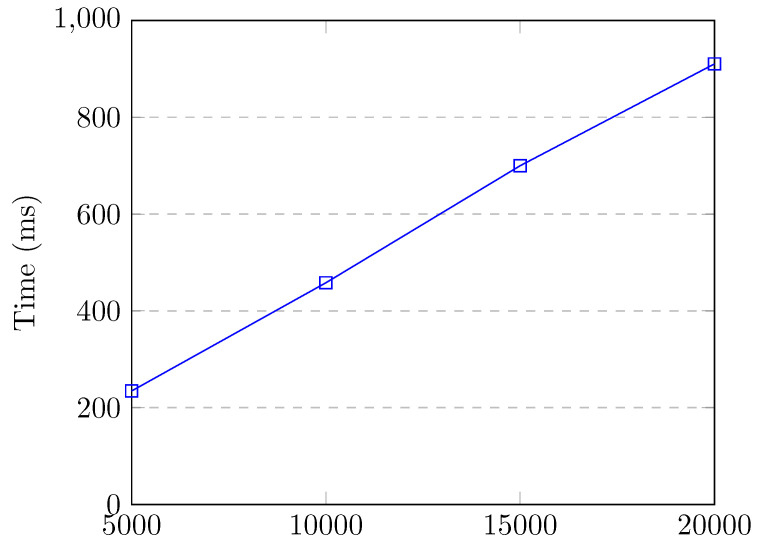
Feature retrieval time of 10 best features and classification up to 20,000 flow entries.

Figure 15 shows the feature retrieval times of the 10 best features and classification time for up to 1000 flow entries in the switch. It is fairly low and it does not affect the performance of the network. Figure 16 shows the feature retrieval times of the 10 best features and classification time for up to 20,000 flow entries in the switch, which corresponds to the DoS attack with spoofed addresses. Feature retrieval and classification take around 900 milliseconds for 20,000 flow entries. However, the SDN application does not wait to finish classifying every flow entry in the switch before taking action. The attackers are blocked immediately when they reach the detection threshold. Therefore, most of the time, attacks are mitigated before a huge number of attack flows are installed into the switch.

Our application calculated the feature vectors and classified them in nine milliseconds when the switch had 100 flow entries. This procedure takes 49 milliseconds when the switch has 1000 flow entries. We believe that these times are fairly low and they do not affect normal operation of the controller and the network. Under a DoS attack, feature vector calculation and classification take less than a second for all 20,000 flow entries. The flow entries are classified one-by-one and mitigation is performed immediately upon attack detection. Therefore, attacks are swiftly mitigated before they can cause serious damage to the target and the network.

#### 6.2.2. Bandwidth Measurements

The maximum available bandwidth of all the links between the switch and hosts in our network were set to 100 Mb per second. The iPerf3 tool [44] was used to measure the available bandwidth between one of the IoT devices and the server with and without the presence of DoS attacks. One malicious host was used to perform a DoS attack targeting the server. The packet sending rate was 1000 packets per second and the payload of the packets was 1000 bytes. Attacks started after five seconds.

**Figure 17:**
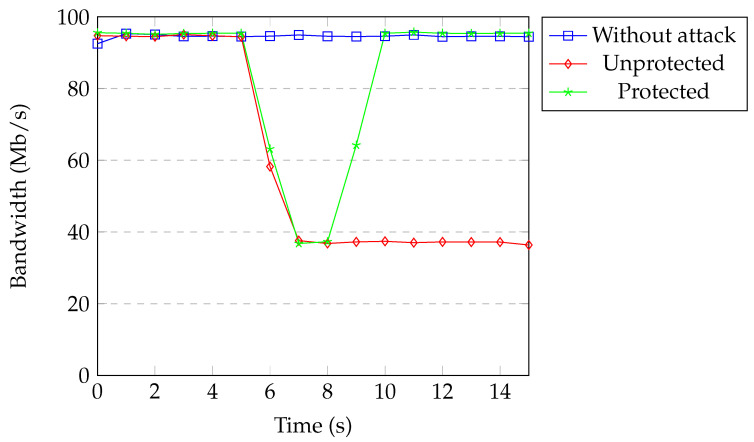
Available bandwidth under SYN flood without spoofing.

Figure 17 shows the available bandwidth under TCP SYN flood attack without spoofing. All of the packets coming from the attacker passed over the same flow entry in the case of no spoofing. Therefore, it took three detection processes to exceed the threshold. Available bandwidth between one of the IoT devices and the server was around 95 Mb per second during the normal operation of the network. Without protection, the bandwidth decreased to 37 Mb per second. When our protection was active, the attacker was blocked based on the physical port after exceeding the threshold. Bandwidth returned back to normal after a couple of seconds.

**Figure 18:**
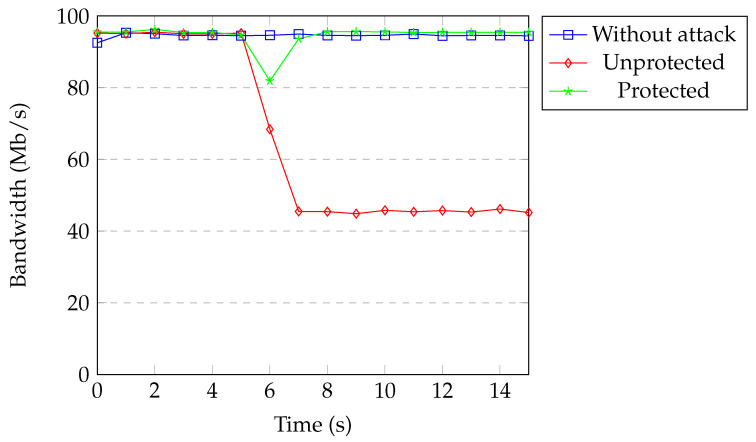
Available bandwidth under SYN flood with spoofing.

Figure 18 shows the available bandwidth under TCP SYN flood attack with spoofing. Every packet coming from the attacker that missed the flow rules in the switch caused a new flow rule installation. This process slowed the forwarding of malicious packets. The available bandwidth under attack decreased to 45 Mb per second. When our protection was active, bandwidth decreased to 82 Mb per second only for a second and then returned back to normal. The threshold was exceeded in the first detection process and the attacker was blocked immediately.

**Figure 19:**
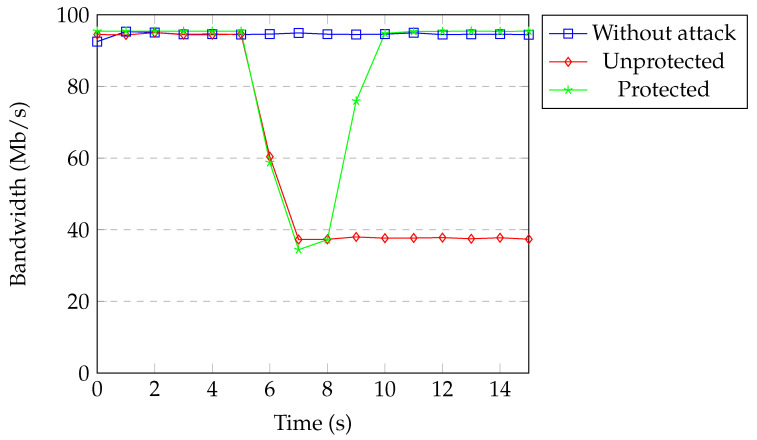
Available bandwidth under UDP flood without spoofing.

**Figure 20:**
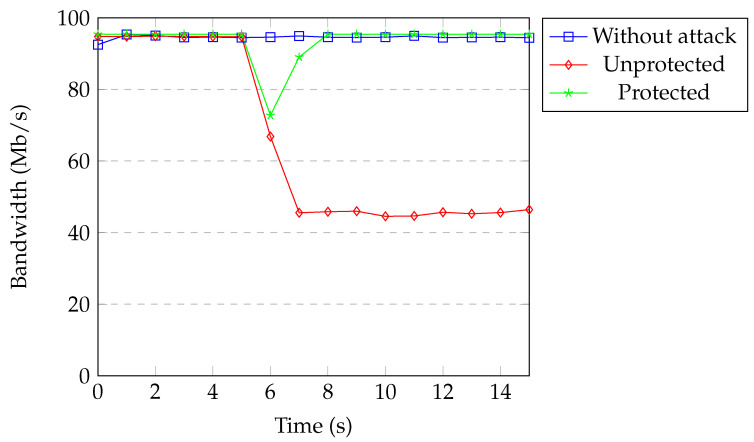
Available bandwidth under UDP flood with spoofing.

Figure 19 and Figure 20 show the available bandwidth under UDP flood attack with and without spoofing. The results are similar to the TCP SYN flood attack.

Overall, the available bandwidth returned back to normal within one to three seconds, depending on the attack properties when our protection was active. Our protection quickly prevents attackers from causing damage to the target and networks. For the DoS attacks with spoofed addresses, attackers are detected within a second and the network recovers immediately. For the DoS attacks without spoofed addresses, our application waits until attackers reach the threshold (around three seconds) and then blocks the attackers. The network recovers in 1–2 s after detection.

#### 6.2.3. CPU Measurements

DoS and DDoS attacks with spoofed addresses waste the resources of both the controller and switches. Spoofed match fields of the attack packets cause “table miss” events for each packet. Switches buffer these packets and send a packet_in message to the controller for every attack packet. The controller processes these packets and decides the route. The controller sends a packet_out message to the switch, which contains the determined action for the packet. The controller also installs flow rules for every attack packet.

The ONOS controller and switch were running on the same machine in these experiments. Therefore, we used the Linux top command to measure CPU usages of their processes. Our machine had six cores and two threads per core, which makes the maximum CPU utilization 1200%. One malicious host was used to perform the DoS attack with spoofed IP addresses. Packet sending rate was 1000 packets per second and payload of the packets was 0 byte. Attacks started after five seconds. Under normal conditions, most of the time the CPU utilization of the hosts were 0%. Rarely, CPU utilization of the two benign hosts that sent data to the server were 5.9–6.1%. During DoS attacks, the CPU utilization of the attacker was around 30%.

**Figure 21:**
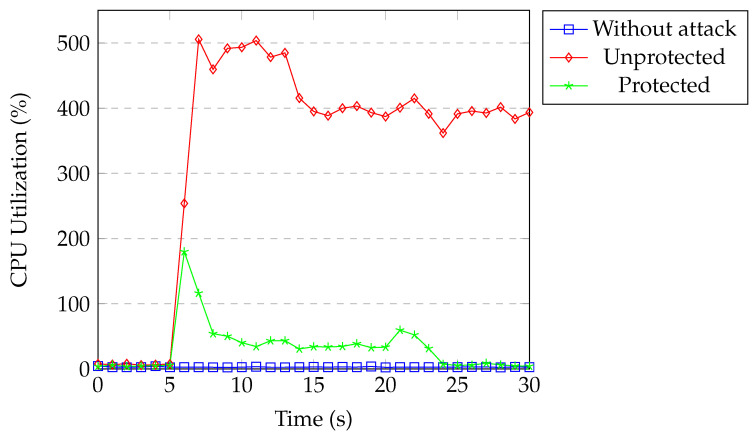
Controller CPU under SYN flood.

Figure 21 shows the CPU usage of the controller under TCP SYN flood attack. CPU usage was around 2% for our normal network traffic. During the attack without protection, CPU utilization reached 500% within two seconds and stayed there for 7–8 s. Subsequently, it dropped to 400%. When our protection was active, CPU utilization reached 180% for a second and then dropped to around 35% for the next 15 s. Afterwards, CPU utilization returned to normal. Even though the attacker was blocked in the first detection process, attack flows were installed into the switch until the controller installed the block rule. Our classification model kept classifying them in the following detection processes until these flow entries timed out. This is the reason why CPU utilization remained around 35% for a short time.

**Figure 22:**
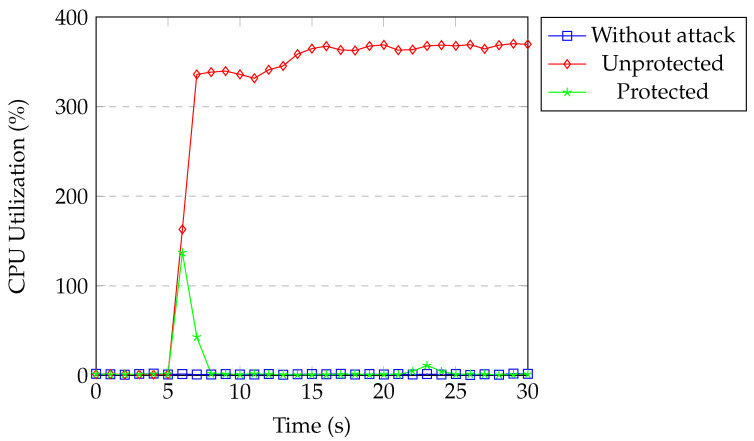
Switch CPU under SYN flood.

Figure 22 shows the CPU usage of the switch under TCP SYN flood attack. The CPU usage was around 1% for our normal network traffic. During the attack without protection, the CPU utilization of the switch process reached around 370%. When our protection was active, CPU utilization increased to 135% for a second and then returned back to normal within a couple of seconds. The controller installed the flow block rule in the first detection process and all of the packets coming from the attacker were dropped by matching the installed flow rule.

**Figure 23:**
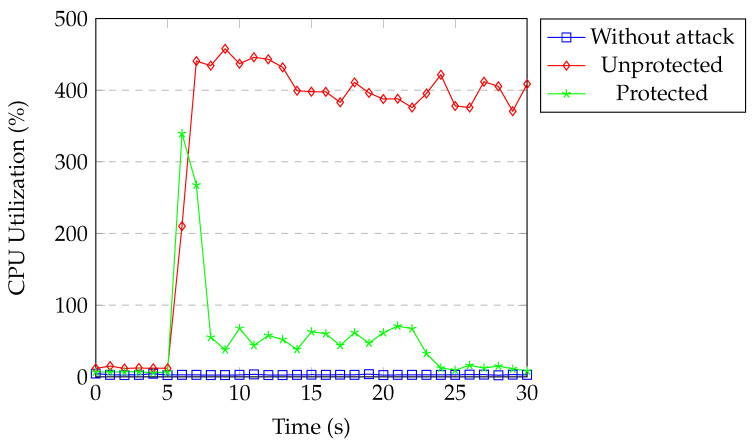
Controller CPU under UDP flood.

**Figure 24:**
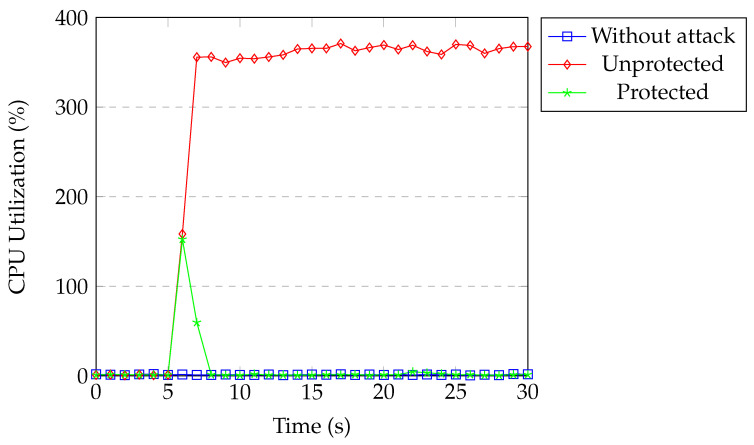
Switch CPU under UDP flood.

Figure 23 and Figure 24 show the CPU utilization of the controller and the switch. The results are similar to the TCP SYN flood attack experiments.

Overall, without our protection, both the controller and switch consumed around 400% of the CPU, 800% total. The DoS attack wasted a huge part of the CPU of the switch and the controller when considering the maximum CPU utilization of our machine was 1200%. One attacker caused the network to use 2/3 of its available CPU. When we performed the DDoS attack with four attackers, CPU utilization reached to a maximum in a short time and the SDN controller crashed after some time. When our protection was active, attacks were detected within a second and the attackers were blocked immediately and the switch’s CPU utilization went back to normal, which is close to 2%. All of the packets coming from the attacker matched with the block rule and were dropped. Our application kept classifying attack rules remaining in the switch until they timed out. Therefore, the CPU utilization of the controller was around 40% for 15–20 s after attack detection. Subsequently, CPU utilization returned back to normal.

## 7. Conclusions

In this work, we proposed an automated, intelligent intrusion detection and mitigation approach for SDN, which aims to provide explainable security in the IoT networks of the 5G era. The proposed approach relies on automated flow feature extraction and highly accurate classification of network flows by a random forest classifier in the SDN application layer, for detecting various classes of attacks and taking remedial action through the installation of new flow rules with high priority at the data plane. We presented our SDN-specific dataset modeling a realistic IoT environment, which includes flow data for common network attacks as well as normal traffic, and provided results on the accuracy of intrusion detection as well as performance results in the presence and absence of our proposed security mechanism.

The proposed security approach is promising for achieving real-time, highly accurate detection and mitigation of attacks in SDN-managed networks, which will be in widespread use in the 5G and beyond era. We believe that the created dataset will also be a useful resource for further research in ML-based intrusion detection in SDN-managed IoT networks. Our future work will include an extension of the created dataset with more attack types and network topologies, as well as an evaluation of the proposed security approach with these additional network conditions. We also aim to integrate an interface for interpretability by human experts to further enhance the explainability of the security model. While the proposed approach has achieved successful results in the network environment it has been trained for, applicability to different networks will require training the model actively through online learning. This will not only provide the capability to detect previously detected attack types, but also to correctly classify recently arising attacks through continuous learning. While transfer learning approaches are successful to a certain extent, their performance cannot compete with the performance of training ML models with datasets being obtained in the real operation environment in many cases. Therefore, our future work will also focus on building a continuous learning with human-in-the-loop system, which is expected to be achieve high performance in a variety of network structures.

## Figures and Tables

**Table 1:** Flow feature descriptions.

Feature	Description
Dur	Record total duration
Mean	Average duration of aggregated records
Stddev	Standard deviation of the duration of aggregated records
Spkts	Source-to-destination packet count
Sbytes	Source-to-destination byte count
Dbytes	Destination-to-source byte count
Sum	Total duration of aggregated records
TnP_PSrcIP	Total number of packets per source IP
TnP_PDstIP	Total number of packets per destination IP
TnP_Per_Dport	Total number of packets per destination port

**Table 2:** Features of the created SDN datasets.

Feature	Description
srcMac	Source MAC address
dstMac	Destination MAC address
srcIP	Source IP address
dstIP	Destination IP address
srcPort	Source port number
dstPort	Destination port number
last_seen	Record last time
Protocol	Textual representation of network protocol
proto_number	Numerical representation of network protocol
Dur	Record total duration
Mean	Average duration of aggregated records
Stddev	Standard deviation of the duration of aggregated records
Min	Minimum duration of aggregated records
Max	Maximum duration of aggregated records
Pkts	Total count of packets in transaction
Bytes	Total number of bytes in transaction
Spkts	Source-to-destination packet count
Dpkts	Destination-to-source packet count
Sbytes	Source-to-destination byte count
Dbytes	Destination-to-source byte count
Srate	Source-to-destination packets per second
Drate	Destination-to-source packets per second
Sum	Total duration of aggregated records
TnBPSrcIP	Total number of bytes per source IP
TnBPDstIP	Total number of bytes per destination IP
TnP_PSrcIP	Total number of packets per source IP
TnP_PDstIP	Total number of packets per destination IP
TnP_PerProto	Total number of packets per protocol
TnP_Per_Dport	Total number of packets per destination port
N_IN_Conn_P_SrcIP	Number of inbound connections per source IP
N_IN_Conn_P_DstIP	Number of inbound connections per destination IP
Attack	Attack or not
Category	Traffic category

**Table 3:** Distribution of records in the first SDN dataset.

Category	Size (M)	%
Normal	1.67	5.99
DoS	0.79	2.84
DDoS	0.19	0.67
Port Scanning	20.68	74.08
OS and Service Detection	3.39	12.15
Fuzzing	1.18	4.24

**Table 4:** Distribution of records in the second SDN dataset.

Category	Size (M)	%
Normal	2.67	8.84
DoS	0.49	1.67
DDoS	0.18	0.60
Port Scanning	22.44	74.23
OS and Service Detection	3.39	11.20
Fuzzing	1.05	3.48

**Table 5:** Initially selected features.

Feature	Description
Dur	Record total duration
Mean	Average duration of aggregated records
Spkts	Source-to-destination packet count
Sbytes	Source-to-destination byte count
Dbytes	Destination-to-source byte count
Sum	Total duration of aggregated records
TnP_PSrcIP	Total number of packets per source IP
TnP_Per_Dport	Total number of packets per destination port
N_IN_Conn_P_SrcIP	Number of inbound connections per source IP
N_IN_Conn_P_DstIP	Number of inbound connections per destination IP

**Table 6:** Performance of random forest (RF) using initially selected 10 features.

Class	F1 Score	Precision	Recall
Normal	94.69	96.71	92.75
DoS	98.37	98.08	98.66
DDoS	98.03	97.21	98.87
Port scanning	98.86	98.54	99.19
OS and service detection	98.36	98.35	98.37
Fuzzing	98.86	98.32	99.41

**Table 7:** 10 best features.

Feature	Description
Dur	Record total duration
Mean	Average duration of aggregated records
Stddev	Standard deviation of aggregated records
Spkts	Source-to-destination packet count
Sbytes	Source-to-destination byte count
Dbytes	Destination-to-source byte count
Sum	Total duration of aggregated records
TnP_PSrcIP	Total number of packets per source IP
TnP_PDstIP	Total number of packets per destination IP
TnP_Per_Dport	Total number of packets per destination port

**Table 8:** Hyperparameters of Random Forest model.

Hyperparameter	Value
n_estimators	100
criterion	entropy
max_depth	40
max_features	auto
min_samples_leaf	1
min_samples_split	6
bootstrap	True
random_state	0

**Table 9:** Performance of random forest (RF) using 10 best features.

Class	F1 Score	Precision	Recall
Normal	94.80	96.75	92.92
DoS	98.44	97.77	99.12
DDoS	97.93	97.29	98.58
Port scanning	98.89	98.52	99.27
OS and service detection	98.08	98.40	97.76
Fuzzing	98.76	98.20	99.32
